# Variation in Genes Related to Cochlear Biology Is Strongly Associated with Adult-Onset Deafness in Border Collies

**DOI:** 10.1371/journal.pgen.1002898

**Published:** 2012-09-13

**Authors:** Jennifer S. Yokoyama, Ernest T. Lam, Alison L. Ruhe, Carolyn A. Erdman, Kathryn R. Robertson, Aubrey A. Webb, D. Colette Williams, Melanie L. Chang, Marjo K. Hytönen, Hannes Lohi, Steven P. Hamilton, Mark W. Neff

**Affiliations:** 1 Department of Psychiatry and Institute for Human Genetics, University of California San Francisco, San Francisco, California, United States of America; 2 Institute for Human Genetics, University of California San Francisco, San Francisco, California, United States of America; 3 Neurogenomics Division, The Translational Genomics Research Institute, Phoenix, Arizona, United States of America; 4 Veterinary Genetics Laboratory, University of California Davis, Davis, California, United States of America; 5 CullenWebb Animal Neurology and Ophthalmology Centre, Riverview, New Brunswick, Canada; 6 Department of Clinical Neuroscience, Faculty of Medicine, University of Calgary, Calgary, Alberta, Canada; 7 School of Veterinary Medicine, University of California Davis, Davis, California, United States of America; 8 Department of Anthropology, University of Oregon, Eugene, Oregon, United States of America; 9 Department of Veterinary Biosciences and Research Programs Unit, Molecular Medicine, University of Helsinki and Folkhälsan Research Center, Helsinki, Finland; 10 The Van Andel Research Institute, Grand Rapids, Michigan, United States of America; Cornell University, United States of America

## Abstract

Domestic dogs can suffer from hearing losses that can have profound impacts on working ability and quality of life. We have identified a type of adult-onset hearing loss in Border Collies that appears to have a genetic cause, with an earlier age of onset (3–5 years) than typically expected for aging dogs (8–10 years). Studying this complex trait within pure breeds of dog may greatly increase our ability to identify genomic regions associated with risk of hearing impairment in dogs and in humans. We performed a genome-wide association study (GWAS) to detect loci underlying adult-onset deafness in a sample of 20 affected and 28 control Border Collies. We identified a region on canine chromosome 6 that demonstrates extended support for association surrounding SNP Chr6.25819273 (p-value = 1.09×10^−13^). To further localize disease-associated variants, targeted next-generation sequencing (NGS) of one affected and two unaffected dogs was performed. Through additional validation based on targeted genotyping of additional cases (n = 23 total) and controls (n = 101 total) and an independent replication cohort of 16 cases and 265 controls, we identified variants in *USP31* that were strongly associated with adult-onset deafness in Border Collies, suggesting the involvement of the NF-κB pathway. We found additional support for involvement of *RBBP6*, which is critical for cochlear development. These findings highlight the utility of GWAS–guided fine-mapping of genetic loci using targeted NGS to study hereditary disorders of the domestic dog that may be analogous to human disorders.

## Author Summary

The domestic dog offers a unique opportunity to study complex disorders similar to those seen in humans, but within the context of the much simpler genetic backgrounds of pure breeds, which represent closed populations. We performed a whole-genome search for genetic risk factors of adult-onset deafness in the Border Collie, a breed of herding dog that relies on acute hearing to perceive and respond to commands while working. Adult-onset deafness in Border Collies typically begins in early adulthood and is similar to age-related hearing loss in humans. This earlier onset has particular impact on the utility of working Border Collies and the livelihoods of their owners, and it appears to have a genetic cause. We identified three genetic variants that were strongly associated with adult-onset deafness in a sample of 405 Border Collies. These variants are located in two genes that have previously been linked to deafness, one involved in ear development and another that appears to mitigate tissue damage in the ear. These results provide new insight regarding genetic risk factors for age-related hearing loss in both dogs and humans.

## Introduction

Age-related hearing loss (presbycusis) occurs in humans with a prevalence of about 40% in individuals older than 65 years of age. It is associated with difficulties of communication, isolation, depression and possibly even dementia in the severely affected [1]. There are extensive genetic contributions to hearing variation [2], which has an estimated heritability of 35–55% (reviewed in [3]). Studies in humans have identified risk-conferring variants in both mitochondrial [4], [5] and autosomal DNA (reviewed in [3]). A recent genome-wide association study (GWAS) performed in an isolated Finnish population identified the candidate gene *IQ motif containing GTPase activating protein 2 (IQGAP2)* as a novel risk locus for hearing loss [6], as well as modest support for another, previously identified GWAS candidate, *metabotropic glutamate receptor 7 (GRM7)* [7]. Overall, however, the breadth of genetic variation that may confer risk for this common disorder remains unknown.

The domestic dog offers a unique opportunity to explore the genetic backgrounds of naturally occurring disorders that are analogous to human diseases. Genomic studies are particularly informative when a disorder of interest demonstrates a simpler inheritance pattern in dogs than in humans, suggesting one or a few main risk alleles. Deterioration of hearing with age is normal in dogs, with an onset at 8–10 years [8] that corresponds with physiological changes in critical systems in the ear, including reduced spiral ganglion neuronal density in the cochlea [9]. Shimada et al. [10] reported that dogs with hearing loss demonstrated the same four types of lesions found in humans (as described by Schuknecht & Gacek [11]): sensory, neural, strial and cochlear conductive lesions. Physiological measurements of hearing ability using brainstem auditory evoked response (BAER) demonstrate similar patterns in dogs and humans, with high- and mid-range frequencies being the most severely affected [8], [12]. Thus, age-related hearing loss may be similar in both clinical presentation and underlying pathology in humans and dogs.

Across breeds, presbycusis is estimated to begin at 8–10 years, when deterioration is observed at all frequencies [8]. However, adult-onset deafness in Border Collies often has an earlier onset (3–5 years) than deafness resulting from the physiological aging of hearing organs. Distinct from other breeds, the Border Collie has been selected for over 100 years to perceive and respond to whistle commands while working at distances of 800 meters or more from a handler. Being able to detect slight differences in whistle tones is essential to the function of a working Border Collie, and even moderate hearing loss in one ear can have a major impact on working ability. Although relatively uncommon in Border Collies, adult-onset deafness is considered especially problematic because hearing is so integral to the tasks for which these dogs are selectively bred and used. In addition, dogs afflicted by adult-onset deafness are often in their prime working years, with the average age of top working dogs around 7 years [13].

The earlier age of onset in affected Border Collies suggests that adult-onset deafness is genetically influenced and possibly more severe than that observed in other breeds of dogs. Many of the affected dogs included in this study were reported by their owners to have one or more first-degree family members with similar deafness. We undertook a study to identify genetic risk factors and address concerns regarding adult-onset deafness among Border Collies, as well as potentially gain information about analogous human conditions.

## Results

### Adult-Onset Deafness in Border Collies

The exact age of onset of hearing deterioration is often difficult to ascertain in pet dogs, because subtle changes may go unnoticed by pet owners and because dogs are known to compensate for hearing loss [14]. However, the owners of the dogs included in this study estimated the age of onset of hearing loss based upon close observations of behavioral characteristics in working dogs indicating poor hearing (e.g., reduced call distance, poor performance). The average estimated age of onset was 4.3 years (S.E. of 0.5 years), with a range of 1–9 years.

### Genome-Wide Association Study

**Figure 1:**
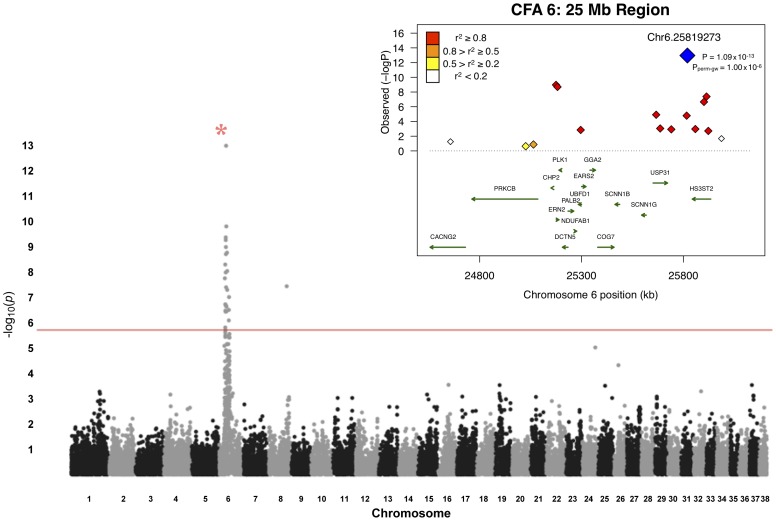
Manhattan plot of GWAS for adult-onset deafness. Chromosome markers are plotted on the x-axis in order and alternately shaded. The -log_10_(p-value) is plotted on the y-axis. The red line indicates significance at the Bonferroni-corrected level for 30,000 SNPs. There is extensive regional support for an association on CFA6. The inset shows an enlargement of the 25-Mb association region, including genes of interest. The raw GWAS and permuted p-values (P_perm-gw_) for the top SNP are also given.

A total of 48 unrelated Border Collies (20 cases, 28 controls) were utilized for the primary association study (Table S1). Following quality control of the genotype data, 30,231 SNPs were retained for genetic mapping. Genome-wide association analyses with EMMAX identified a region on CFA6, at approximately 25 Mb (Figure 1). In total, 25 markers exhibited significance beyond a Bonferroni-corrected threshold (p = 1.65×10^−6^ for 30,231 tests). The strongest finding was an intergenic SNP, Chr6.25819273, with a p-value of 1.09×10^−13^ (Table 1), with strong regional support demonstrated by neighboring SNPs whereby, within 1 Mb flanking the top finding, six reached Bonferroni significance. The closest predicted gene to this SNP is *HS3ST2*, approximately 24 kb downstream of the marker. HS3ST2 is a member of the heparan sulfate biosynthetic enzyme family, and is expressed predominantly in the brain [15].

**Table 1:** Top 25 ranked findings from analysis for presbycusis in Border Collies.^a^

SNP	A1/A2	Freq_Case_	Freq_Control_	P_EMMAX_	P_Allelic_	P_Perm_	OR (95% CI)
Chr6.25819273	C/A	0.00	0.78	1.09E-13	6.42E-14	1.00E-06	N/A
Chr6.26517587	A/G	0.83	0.14	1.64E-10	2.71E-11	1.00E-06	28.29 (9.35–85.57)
Chr6.24591869	T/C	0.10	0.77	4.46E-10	1.09E-10	2.00E-06	0.034 (0.01–0.11)
Chr6.24577002	C/T	0.89	0.20	5.68E-10	2.91E-11	1.00E-06	34.77 (10.18–118.7)
Chr6.25174415	C/G	0.10	0.78	1.07E-09	8.06E-11	2.00E-06	0.03 (0.01–0.11)
Chr6.28753894	A/G	0.78	0.13	1.78E-09	1.36E-10	3.00E-06	24.11 (8.15–71.38)
Chr6.25181733	G/A	0.89	0.21	2.03E-09	9.38E-11	2.00E-06	31.17 (9.23–105.20)
Chr6.22844453	G/A	0.80	0.19	5.21E-09	3.07E-09	2.60E-05	17.6 (6.25–49.56)
Chr6.29363433	T/G	0.78	0.15	9.45E-09	1.07E-09	6.00E-06	19.81 (6.89–56.92)
Chr6.24570819	T/G	0.10	0.75	1.07E-08	3.28E-10	4.00E-06	0.037 (0.01–0.12)
Chr6.21475826	C/T	0.80	0.23	1.83E-08	3.87E-08	4.20E-04	13.23 (4.90–35.7)
Chr8.62484232	T/G	0.00	0.37	3.75E-08	1.44E-05	1.70E-01	N/A
Chr6.25913101	C/T	0.00	0.61	4.14E-08	8.67E-10	5.00E-06	N/A
Chr6.29470484	T/C	0.76	0.15	5.20E-08	3.15E-09	2.60E-05	18.53 (6.42–53.46)
Chr6.35491820	G/C	0.50	0.04	1.00E-07	2.68E-07	2.82E-03	25.00 (5.34–117.00)
Chr6.23160353	C/A	0.53	0.05	1.95E-07	1.45E-07	1.57E-03	19.53 (5.23–72.98)
Chr6.23166082	G/A	0.53	0.05	1.95E-07	1.45E-07	1.57E-03	19.53 (5.23–72.98)
Chr6.25900591	G/A	0.00	0.54	2.21E-07	2.37E-08	2.21E-04	N/A
Chr6.26959216	C/A	0.03	0.61	2.40E-07	5.15E-09	3.60E-05	0.02 (0.002–0.13)
Chr6.34915222	G/A	0.63	0.14	3.21E-07	9.41E-07	1.18E-02	10.00 (3.74–26.77)
Chr6.23177930	C/T	0.53	0.06	3.48E-07	2.46E-07	2.57E-03	18.79 (5.02–70.3)
Chr6.26917473	C/A	0.03	0.59	3.67E-07	1.20E-08	9.50E-05	0.02 (0.002–0.14)
Chr6.24104844	A/G	0.08	0.66	3.76E-07	9.54E-09	7.30E-05	0.04 (0.01–0.15)
Chr6.34819558	A/T	0.03	0.38	8.32E-07	5.76E-05	5.31E-01	0.04 (0.005–0.33)
Chr6.22861769	T/A	0.50	0.05	1.58E-06	4.37E-07	4.99E-03	17.67 (4.73–66)

a SNP: marker name (location information); A1: risk allele; A2: reference allele; Freq_Case_: allele frequency of A1 in cases; Freq_Control_: allele frequency of A1 in controls; P_EMMAX_: p-values from EMMAX primary GWAS; P_Allelic_: p-values from allelic association analysis; P_Perm_: genome-wide (EMP2) permuted p-values from PLINK; OR (95% CI): odds ratio with 95% confidence interval as calculated with logistic regression.

All top 25 hits from the EMMAX analysis reached statistical significance at the Bonferroni-corrected level, and all but one are on CFA6. All top 25 findings also reached genome-wide significance after empirical significance testing with permutation. Odds ratios calculated in PLINK demonstrate strong effects for all top hits.

Associations were also assessed through permutation analysis in PLINK. One million permutations yielded genome-wide permutated p-values that achieved genome-wide significance (Table 1). Analyses of copy number variation using genome-wide SNP data did not reveal evidence of structural changes associated with hearing loss. Association modeling suggested an autosomal recessive mode of inheritance for adult-onset deafness in Border Collies.

### Fine-Mapping

The large candidate region identified on CFA 6 was syntenic with human 16p12.1-p12.3, which encompasses the human autosomal recessive deafness locus *DFNB22* [16]. A candidate of immediate interest within this region was the gene *OTOA*, defects of which were implicated in a case of prelingual sensorineural deafness in a consanguineous Palestinian family [16]. We performed PCR amplifications of the 28 exons and a highly conserved non-coding region. PCR products were sequenced and analyzed for mutations in affected dogs. None of the observed polymorphisms tracked specifically in affected dogs.

**Figure 2:**
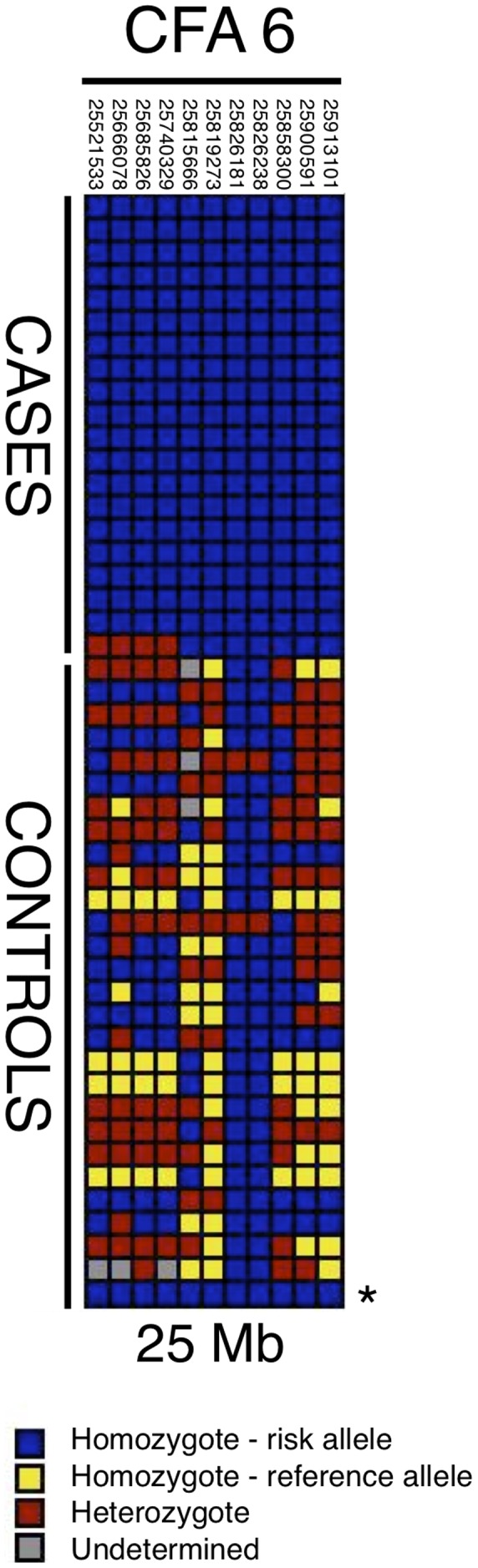
Haplotypes in CFA6 region 25 Mb. Each box color represents a different genotype, as indicated by the key; dogs are listed in rows and SNPs in columns. Case dogs are all homozygous for a single haplotype spanning 7 markers, and all but one case also share an 11-SNP haplotype (for which the single dog is heterozygous). One sample used as a control (marked with *) also carries the 11-SNP risk haplotype.

Given the large region of association and lack of polymorphisms in the strong candidate gene *OTOA*, we next narrowed the critical region for the 25-Mb locus by haplotype analysis. We detected a 7-SNP haplotype that was homozygous in all cases but only once among the control samples (see *Discussion*). A larger 11-SNP haplotype was found homozygous in 19 of 20 cases and present in the same control. (Figure 2). For sequencing via target capture and next-generation sequencing (NGS), we selected an affected dog that was homozygous for the extended 11-SNP risk haplotype at 25 Mb (Figure 2). Two control dogs that did not carry the candidate risk haplotype were also sequenced after target capture by NGS.

The risk haplotype spanning the coordinates on CFA6 from 25.5–25.9 Mb was used to guide mutation discovery with the NGS data. We identified predicted genes based on synteny and the annotated gene index, and designed a solution-based target capture mixture to target exons and introns, along with at least 1 kb of upstream and downstream regions possibly inclusive of UTR sequence. This capture design encompassed 2.3 Mb, and included 73 genes. NGS rendered over 30 million reads per sample (Table S2). More than 90% of these reads could be aligned to the reference genome sequence (CanFam2). Of the total targeted sequence, 75% had greater than 10× coverage, and nearly 70% had >30× coverage (Table S7).

The numbers and types of variants identified are summarized in Table S3. One strong non-synonymous SNP (nsSNP) candidate, Chr6.25714052, is located in exon 17 of *USP31*, which encodes an ubiquitin specific peptidase. It is an A>G variant that is predicted to cause an I847V change in the resulting protein product. The position is highly conserved, with a phastCons score of 0.95 (Table S4), although SIFT predicted the change to be tolerated (SIFT score of 0.66). Also of note in *USP31* is an intronic T>G SNP (Chr6.25681850) that is very highly conserved (phastCons score of 0.98) and is 5 bp away from an intron-exon boundary. This variant was called G/G in the case and T/T in both controls. Both variants are located within the risk haplotype.

Another candidate nsSNP, Chr6.24500625, is in exon 18 of *RBBP6*, encoding a retinoblastoma binding protein. This nsSNP changes threonine to asparagine at residue 1,397. This G>T variant was called T/T in the case and G/G in both controls. SIFT predicted the change to be tolerated (SIFT score = 0.69; Table S4). Although the conservation score for this SNP is low (phastCons = 0.001) and the variant is located upstream of the main risk haplotype, RBBP6 (also known as PACT) plays a critical role in ear development and hearing; disruption of the gene has been shown to cause congenital hearing impairment in mice [17] and suggests high relevance to hearing loss in dogs. The sequencing data from 25 Mb did not exhibit variants in *OTOA* that were homozygous in the case but not in controls. Therefore, we did not consider this to be the causative gene. Although small insertion/deletion variants were found in the mapped intervals, none of these variants appeared to be causal.

The three variants described above, Chr6.24500625 in *RBBP6*, and Chr6.25681850 and Chr6.25714052 in *USP31*, were the most compelling for follow-up genotyping analyses due to biological implications (*RBBP6*) and location within the risk haplotype (*USP31*), and were analyzed both for validation (primary mapping cohort) and replication (independent cases and controls).

### Validation

Genotyping was performed via dye-terminator sequencing for the three chosen variants. All three showed associations with adult-onset deafness (Table 2). For replication analysis, we genotyped an independent Border Collie cohort of 16 cases and 265 controls. All three SNPs were strongly associated with adult-onset deafness (Table 2), replicating our previous mapping results. Meta-analysis of the combined primary and replication cohorts yielded even stronger associations for all three variants. The strongest association was found for the variant of *USP31*, Chr6.25681850, with p = 6.16×10^−22^ (Table 2).

**Table 2:** Summary of association results with combined data from all stages of study.^a^

			Primary			Replication			
SNP	Gene	A1/A2	Fr_Case_/Fr_Cont_	P	OR	Fr_Case_/Fr_Cont_	P	OR	Comb. P
			n = 23/n = 101			n = 16/n = 265			
Chr6.24500625	*RBBP6*	T/G	0.91/0.38	1.98E–10	16.32	0.69/0.41	0.0019	3.20	1.01E–9
					(5.62–47.41)			(1.49–6.89)	
Chr6.25681850	*USP31*	G/T	0.98/0.31	3.24E–16	97.98	0.72/0.23	8.57E–10	8.55	6.16E–22
					(13.19–727.90)			(3.85–18.96)	
Chr6.25714052	*USP31*	G/A	0.98/0.74	0.0012	13.52	0.91/0.71	0.016	3.96	0.00015
					(1.81–100.90)			(1.19–13.19)	

a SNP: marker name (location information); A1: risk allele; A2: reference allele; Fr_Case_: allele frequency of A1 in cases; Fr_Cont_: allele frequency of A1 in controls; P: p-values from allelic association analysis; OR: odds ratio with 95% confidence interval; Comb. P: combined p-value from meta-analysis.

The strength of association of the three variants is shown. Chr6.24500625 in *RBBP6* and Chr6.25681850 in *USP31* remain highly significant after inclusion of more cases and controls.

## Discussion

Our results represent the first GWAS of adult-onset deafness in the domestic dog. We demonstrated the successful application of target capture for next-generation sequencing (NGS) in the dog. The region implicated by GWAS in our study is syntenic to regions implicated in congenital sensorineural deafness in humans.

In this study, we identified three strong candidate coding and non-coding variants associated with adult-onset deafness. The strongest is Chr6.25681850, an intronic SNP in *USP31* that is 5 bp from an intron-exon boundary and may play a role in alternate splicing (as annotated in humans). Preliminary studies of mRNA collected from peripheral blood samples from two dogs harboring this variant did not suggest changes in RNA splicing in this region, though tissue-specific changes in RNA regulation cannot be ruled out. *USP31* is a ubiquitin-related gene that has been linked to Parkinson's disease in humans [18]. The implication of a ubiquitin-related gene in adult-onset deafness is particularly intriguing given the histological findings of Shimada et al. [10], which included ubiquitin-positive granules in the neuropil of cochlear nuclei of aging dogs. USP31 has also been shown to regulate NF-κB activation; NF-κB deficiency is associated with increased levels of cochlear apoptosis and hearing loss [19], [20]. Despite its location outside the main risk haplotype implicated in the primary GWAS, the second-strongest association was the nsSNP Chr6.24500625, which is exonic to *RBBP6*, a gene previously implicated in hearing in a knockout mouse model [17]. In addition to roles in development, RBBP6 may also be involved in chaperone-mediated ubiquitination and protein quality control [21], suggesting another potential role in pathology. A second *USP31* SNP, Chr6.25714052, was also associated with adult-onset deafness in our cohort, although this locus had the lowest odds ratio of the three candidate loci.

There are several caveats to the present study. A recent human GWAS for presbycusis adjusted phenotypes for hearing thresholds according to age and sex, due to observed variability in hearing threshold in males and females [6]. We elected not to correct for sex in our canine study because such sexual dimorphism is not yet established in aging dogs [8], [10], [12], [14]. Further, we did not adjust for age because the age of onset for our sample cohort, which is likely a specific trait of this form of hearing loss, was owner-estimated. The mean age of our control group was 6.6 years, which is close to the range of hearing loss onset. Therefore, it is possible that dogs categorized as “controls” may, at later stages in life, demonstrate hearing loss similar to that observed in cases. For example, one interesting case involves a dog that was classified as a control at the time of collection (41 months old) and was shown to carry the 11-SNP risk haplotype we identified in affected dogs (this dog is indicated by the asterisk in Figure 2). This dog was later found to have several deaf siblings. In the follow-up SNP genotyping cohort, several Finnish dogs classified as controls by owner questionnaires were also found to carry one or more of the risk alleles identified during NGS (Table 2). Two of these dogs were later found to have had changes in hearing since initial sample collection, and further inquiry uncovered additional family histories of hearing loss in both dogs' pedigrees. However, the misclassification of cases as controls would only reduce analytical power to detect genetic associations, and would not result in spurious associations. Given the strengths of the associations we identified on CFA6, this does not seem to be a concern. Similarly, the presence of the risk haplotype in the homozygous state in all cases suggests that we are not detecting phenotypic heterogeneity influenced by another locus, such as occult congenital unilateral pigmentation-related forms of deafness.

Another caveat stems from the fact that we performed target enrichment for selected regions (i.e., all predicted genes) of extended association loci, and therefore non-coding variants far outside of known or predicted genes were potentially missed. Target enrichment results in uneven coverage, so variants may be missed because not all positions are covered equally well, although the regions that were captured appear to be well assembled (Figures S3, S4; Table S6). Finally, the magnitude of our findings on CFA6 in the primary GWAS (Figure S2) likely overshadowed signals from other regions, even if modifying loci were present.

A strength of canine research highlighted by this study is the reliability of owners to assess phenotype. Each case in the GWAS for adult onset hearing loss was scored by the owner. The results of mapping showed that every case was homozygous for an ancestral risk haplotype, providing compelling support of the initial owner assessment. We view this as tapping the same insights gained by a parent, who develops an intimate awareness of their child's health and behavior. Our results have implications for researchers interested in other canine behavioral traits, in that owner-based observation may be sufficient, at least initially, to advance genetic studies.

Although we observed robust associations and replications, none of the candidate SNPs we identified tracked perfectly with adult-onset deafness. This discrepancy has several possible explanations: 1) adult-onset deafness in the Border Collie is a multigenic trait, 2) the risk locus shows incomplete penetrance, or 3) the variants we identified are in linkage disequilibrium with the true disease-causing mutation. The fact that the *RBBP6* SNP demonstrated a stronger association than the second *USP31* SNP, Chr6.25714052, likely reflects extended linkage in cases that was not readily apparent in haplotype analyses, and may provide information regarding the location of the true causative variant. Given that the 7-SNP homozygous haplotype is present in all cases, it is likely that the variants we identified, which do not track perfectly with larger samples of cases, are more recent in origin than the common tagging SNPs utilized in array genotyping. This would suggest that the causative variant has occurred within the context of a broader, ancestral haplotype. The causative mutation for adult-onset deafness may be a non-coding variant between Chr6.24500625 and Chr6.25681850 that was not captured during target enrichment, and structural variation may also be missed with this technology. Numerous mapping studies in the dog have identified structural variants as causative mutations of traits or disorders [22].

The risk allele of the most strongly-associated SNP from NGS exhibited a frequency of 0.23–0.31 in our Border Collie control sample (Table 2). Future studies may clarify whether this risk allele occurs at similar frequencies in other breeds of dog. Alternative mapping strategies utilizing highly polymorphic microsatellite markers in haplotypes and including different breeds of dog may allow for more refined mapping of structural variants underlying adult-onset deafness. In light of our strong genetic findings, longitudinal studies of dogs that carry risk alleles are warranted for further phenotypic characterization, including histopathologic examination of the middle ears and cochlea. Such investigations may allow us to further characterize and explore the hypothesis that these animals are affected by pure sensorineural deafness, as demonstrated by BAER testing. Observations of the effects of risk variants on aspects of hearing throughout the aging process could provide critical prognostic information for the development of diagnostic or therapeutic tools for use in clinical contexts in both dogs and in humans. It is possible that hearing loss is identified earlier by handlers of dogs for which working ability depends strongly on hearing acuity, such as working Border Collies. Physiological findings may thus be particularly relevant to studies of other utility-bred dogs, in addition to studies of hearing loss that naturally occurs in geriatric dogs.

In conclusion, we identified candidate variants on CFA6 that are strongly associated with adult-onset deafness in Border Collies, with promising implications for future pre-morbid identification of at-risk dogs or applications to human studies. Preliminary causative variant fine-mapping analyses indicate that variants in *USP31* and *RBBP6* may be involved in disease etiology. Future studies to elucidate the roles of these variants in canine adult-onset hearing loss will include haplotype mapping for the detection of structural variations and longitudinal studies of gene effects on hearing electrophysiology trajectories and outcomes.

## Materials and Methods

### Ethics Statement

All work related to animals was performed with the approval of the Institutional Animal Care and Use Program at the University of California, San Francisco (AN079848-02). Collection of blood samples in Finland was approved by the Animal Ethics Committee at the State Provincial Office of Southern Finland (ESLH-2009-07827/Ym-23). The canine samples used were provided by private dog owners, who consented to the use of de-identified data for research purposes.

### Samples

Whole blood samples (3–8 mL) from a total of 48 purebred Border Collies collected in the United States (U.S.) were used for primary GWAS. Samples from 20 affected working Border Collies recruited from owners from the sheepdog/herding community were collected specifically for this genetic survey of risk loci for adult-onset deafness. Twenty-eight control samples (unrelated at the grandparental level, per pedigree analysis) were collected at sheepdog trials or sent directly to the laboratory by owners and breeders in the context of ongoing genetic studies of canine behavior and complex disease. The 20 adult-onset deafness cases included 9 males and 11 females, and the 28 controls included 15 males and 13 females (mean age of controls, 6.6 years). One of the cases and two controls were also sequenced using next-generation sequencing (NGS) technology. An additional 14 U.S. controls and 3 cases and 59 controls collected in Finland were used for follow-up genotyping of candidate variants. Finally, samples from 16 cases and 265 controls were also collected in the U.S. to serve as an independent replication cohort. All follow-up and replication samples were from purebred Border Collies. Although consisting primarily of distinct breeding lines, the Finnish dogs demonstrated similar allele frequencies for the genotyped variants as the U.S. dogs, and thus both groups were analyzed together. The use of a covariate to account for difference in country of origin/breeding line did not change the results of association analysis. DNA was extracted using standard protocols. A summary of the samples is provided in Table S1.

### Phenotypes

Adult-onset deafness phenotypes were assigned based on owner responses to verbal questions to determine whether or not a sampled dog exhibited hearing loss that had developed in adulthood (i.e., deafness that was not congenital). Hearing loss was determined indirectly by owner observations of working dogs that were previously responsive to verbal and whistle commands given in both home and working conditions, but as adults demonstrated significant decreases in response or apparent inability to hear commands. Such loss of hearing was often observed to take place over the course of several months or years. Some owners said that they did not notice any significant changes in their dogs' hearing ability until much later in the dogs' lives, but they suspected that the dog was “compensating” in the work environment by observing the handlers' physical cues or by moving closer to the handler when commands were being given. Controls for the primary GWAS portion of the study were herding Border Collies that met two criteria: 1) genetic clustering in the same group as affected dogs (genetic matching), and 2) no hearing loss indicated in the health sections of behavioral questionnaires completed by owners at the time of sample collection. For follow-up and replication genotyping, U.S. cases were identified as above and controls were defined as dogs that displayed no hearing loss as indicated by owners at the time of sample collection. For all Finnish dogs, deafness phenotypes were obtained through owner interviews via questionnaires.

### Genome-Wide Genotyping

SNP genotyping was performed on the Affymetrix Custom Canine Array v2.0 according to the manufacturer's protocol, a perfect-match-only array targeting 127,000 SNPs (Affymetrix, Santa Clara, CA, USA). Genotypes were called using the BRLMM-P algorithm in Affymetrix Power Tools (apt-1.12.0). Genotype quality control (QC) was first implemented for all of the samples we genotyped on the Affymetrix array for ongoing studies of complex disease (n = 275), which included unrelated dogs as well as a subset of related dogs to assess Mendelian errors. SNP exclusion criteria for the full set were call rates by marker and by individual<95%; concordance of replicate control sample genotypes across all genotyping runs<100%; X-chromosome markers; deviations from Hardy-Weinberg equilibrium with p-values<0.001; minor allele frequency<0.02; and Mendelian errors>5% per SNP. This filtering resulted in a primary dataset of about 40,000 SNPs. After additional QC on only the 48 unrelated samples included in the GWAS for adult-onset deafness (exclusions: SNP call rate<95%, MAF<0.05), approximately 30,000 SNPs were retained for final analysis. QC was performed using Stata10/MP (StataCorp LP, College Station, TX, USA) and PLINK (v1.06–1.07 [23]).

### Target Capture and Next-Generation Sequencing

Genomic library sample preparation was performed using the Illumina single-end library sample preparation kit (Illumina Inc., San Diego, CA, USA). Sample preparation was carried out according to the manufacturer's instructions, except for slight modifications as follows: 3 µg of genomic DNA were sheared via sonication (S-4000 with 2.5″ diameter cup horn, Misonix, Inc., Farmingdale, NY, USA); all purification steps were performed using Agencourt AMPure XP magnetic beads (Beckman Coulter, Inc., Brea, CA, USA); seven cycles of ligation-mediated PCR were used for library amplification. Sample libraries were run on a Bioanalyzer 2100 for DNA quantitation and confirmation of fragment size distribution (High Sensitivity DNA Kit, Agilent Technologies, Waldbronn, Germany). For targeted sequencing, we performed solution-based capture with the Agilent SureSelect Target Enrichment System Kit. Briefly, a custom panel of 120-base cRNA oligos was designed to target 1000 bp upstream and downstream of 73 predicted gene sequences based on mammalian alignments (or in one case, frog) to CanFam2 in the candidate region on CFA6 (Table S6). The target regions were covered by approximately 43,000 probes that were designed for 3× coverage (i.e., each base was covered by three different probes). The prepared genomic libraries were hybridized to the panel of biotin-labeled “bait” oligos for 24 hours. Targets were pulled down via streptavidin magnetic beads, purified, and enriched through 13 cycles of PCR amplification. Samples were single-end sequenced on an Illumina Genome Analyzer IIx for 76 cycles.

### Dye-Terminator Sequencing

Three variants were selected for genotyping via dye-terminator sequencing. All samples included in the study were sequenced. A PCR amplicon was designed for each region, and sequencing was performed in the forward and reverse directions (primer sequences provided in Table S5). We used 1 µL of 1 ng/µL of DNA as input for each standard PCR reaction. Platinum-Taq polymerase was used to amplify segments with a 58°C touchdown protocol in the presence of 0.4 µM primer, 100 µM dNTPs, 2.5 mM Mg and 1 mM betaine.

### Data Analysis

#### GWAS

Primary GWAS analysis was performed using the beta version of Efficient Mixed-Model Association eXpedited (EMMAX) [24]. We used a mixed model-based analysis to account for population stratification or cryptic relatedness that may have been present in the sample (as pedigrees were not available for cases). In addition, allelic associations with one million permutations were performed in PLINK (v1.07, [23]) to further assess association strength and rule out false positives. Permutation analysis consists of reassigning case-control labels randomly (in this case, through one million iterations) and then identifying the distribution of resultant associations at each SNP for all random case-control assignments. This normal distribution of possible associations is then compared to the actual phenotype association; the genome-wide permuted *p*-value represents the probability of seeing a spurious (random) association from the permutation-obtained normal distribution that is stronger than the observed association. For one million permutations, p = 1×10^−6^ states that no random association was stronger than the true finding. In the PLINK analysis, we did not correct for within-breed stratification by the incorporation of principal components or multi-dimensional scaling vectors, since the analyzed dogs had been genetically matched (Figure S1). Finally, haplotype analysis was performed in PLINK, with results visualized using the web-based Genome Variation Server (GVS) (http://gvs.gs.washington.edu/GVS/index.jsp) and Haploview.

#### NGS

Bowtie [25] was used for read alignment against CanFam2, allowing up to 2 mismatches in the first 60 bases of a given read. SAMtools [26], Picard (http://picard.sourceforge.net/), BEDTools [27], and the Genome Analysis Toolkit (GATK, [28]) were used for post-alignment processing. Multi-sample realignment around potential insertion/deletions (indels) and base quality score recalibration were both performed prior to variant calling by GATK's Unified Genotyper. Indels were called using Dindel [29]. ANNOVAR [30] was used to annotate and prioritize variants. Phastcons4way scores, which provide a measure of conservation based on multi-species alignment, were obtained from the UCSC Genome Browser [31].

#### Dye-terminator sequencing

Genotype calls for the three variants were made manually by inspection of sequence traces. Association testing was performed in PLINK, and false positives were assessed by subsequent permutation testing. Allele frequencies of variants between the Border Collies from the U.S. and Finland were not significantly different, according to Fisher's exact test for homogeneity; further, all associations remained when ancestry was added as a covariate (data not shown). The two groups were thus treated as a single group in follow-up analysis.

#### Meta-analysis of candidate variants

A sample size weighted analysis based on p-values generated in the primary and replication cohorts was performed using METAL [32].

## Supporting Information

Figure S1 Multi-dimensional scaling (MDS) vector plots of Border Collies used for deafness analysis. MDS1 x MDS2 based on data from all unrelated Border Collies genotyped by our group for ongoing studies of behavior. A total of 10 MDS covariates were calculated for all Border Collies using a subset of unlinked (r^2^<0.8) whole genome SNP data (∼22 k markers total) in PLINK. Matched controls (blue) were selected based on genetic similarity to cases (red) for the primary GWAS. Samples in gray are shown to demonstrate the overall genetic diversity found in our entire Border Collie cohort.
(PDF)

Figure S2 Q-Q plot of GWAS analysis for adult-onset deafness in Border Collies. Expected versus observed –log_10_(p-value) for the primary GWAS are plotted for each marker; the red line indicates the null distribution. Given the strong association signal by multiple linked markers on CFA6, findings with p-values less than 0.0001 were removed from this plot to avoid skewing the graphical distribution at high observed p-values. The Q-Q plot for this analysis suggests that there is minimal population stratification in this sample, as the majority of points lie on the null distribution.
(PDF)

Figure S3 Number of gaps in the canFam2 assembly from 23 Mb to 29 Mb by size. There were 80 gaps in the canFam2 assembly ranging from 1 bp to 2707 bp (mean = 388 bp; median = 217 bp). The gaps sum to 31089 bp, or about 0.5% of the sequence within the region.
(PDF)

Figure S4 Sequencing assembly quality scores of target capture region in the canFam2 assembly. As shown, most (98%) of the bases in the assembly have quality scores equal or bigger than 40 (deemed high confidence).
(PDF)

Table S1 Sample summary. Samples for the primary genome-wide association study (GWAS) and targeted genotyping were collected from two countries, with breakdown of cases and controls provided for a total of 405 Border Collies.
(DOCX)

Table S2 Next-generation sequencing statistics. Each sample was run in a single lane for 76 sequencing cycles. Given the high number of variants called, we first filtered variants with regard to their genotype in cases and controls, filtering for variants called homozygous in the case sample and called not homozygous for that variant in either of the controls. We then focused on exonic and potentially functional non-coding variants, with priority given to top biological candidates. For a summary of SNPs as annotated in ANNOVAR, see Table S4.
(DOCX)

Table S3 Summary of variants homozygous in case and not in controls using ANNOVAR. Number of SNPs of different locations and functional relevance as annotated by ANNOVAR are provided for total experiment and for which the variants are homozygous in the case sample but not in either control sample (assuming a recessive mode of inheritance as suggested by homozygous risk haplotypes observed in GWAS cases).
(DOCX)

Table S4 Exonic variants for deafness on CFA6. A list of the 26 exonic variants for CFA6 plus annotations is given in Table S4. Gene annotations and predicted amino acid (AA) changes (single letter AA abbreviations flanking AA position) are given with reference to the gene in the human unless the gene is not present in human, in which case it is given for the species noted (Mus – mouse, Sac – yeast, Bos – cow, Rat – rat). Non-synonymous SNPs (nsSNP) are marked in bold. In addition to the called genotypes for each sample, the sequence coverage for that SNP is also provided. Finally, the phastCons4Way score provides a measure of conservation for each position, where values closer to 1 indicate the base is more highly conserved across species. Conservation is based on alignment with human (hg17), mouse (mm6), and rat (rn3). CFA: canine chromosome; Position: base position; Ref: reference allele from genome; Alt: alternate allele observed in sample[s]; genotype: 0 = reference allele, 1 = alternate allele; phastCons score: phastCons4Way score from UCSC genome browser. Of the 26 putative exonic variants, only 8 were annotated to be non-synonymous changes. Four nsSNPs were found in *Abca14*, which was the gene with the most nsSNPs. *Abca14* is an ATP binding cassette transporter gene that has only been annotated in the genomes of rodents [33]. Conservation scores for all four of these nsSNPs were low, suggesting that this gene may not be active and thus tolerant of non-synonymous changes more readily. There was an additional gene containing an nsSNPs, that is not readily linked to hearing function or expression (*EEF2K*).
(DOCX)

Table S5 List of primers used in dye-terminator sequencing. Follow-up sequencing was performed in additional samples for three variants. The PCR conditions used are described in *Materials and Methods*.
(DOCX)

Table S6 List of predicted genes targeted for target capture sequencing and probe coverage by gene. Position of targeted region in canFam2 and number of targets per gene are listed for all target capture regions. An “n/a” is used when a target gene is within another predicted gene that has already been targeted.
(DOCX)

Table S7 Gene coverage information by gene for each target capture sample. Percent bases covered and average coverage depth is provided for each sample that was sequenced using next-generation technology. Coverage is listed by target gene.
(DOCX)

## References

[1] Gates GA, Mills JH (2005) Presbycusis. Lancet 366: 1111–1120 S0140-6736(05)67423-5 [pii];10.1016/S0140-6736(05)67423-5 [doi]. 16182900 10.1016/S0140-6736(05)67423-5

[2] Karlsson KK, Harris JR, Svartengren M (1997) Description and primary results from an audiometric study of male twins. Ear Hear 18: 114–120. 9099560 10.1097/00003446-199704000-00003

[3] Liu XZ, Yan D (2007) Ageing and hearing loss. J Pathol 211: 188–197. 17200945 10.1002/path.2102

[4] Bai U, Seidman MD, Hinojosa R, Quirk WS (1997) Mitochondrial DNA deletions associated with aging and possibly presbycusis: a human archival temporal bone study. Am J Otol 18: 449–453. 9233484

[5] Fischel-Ghodsian N, Bykhovskaya Y, Taylor K, Kahen T, Cantor R, et al (1997) Temporal bone analysis of patients with presbycusis reveals high frequency of mitochondrial mutations. Hear Res 110: 147–154 S0378-5955(97)00077-4 [pii]. 9282897 10.1016/s0378-5955(97)00077-4

[6] Van Laer L, Huyghe JR, Hannula S, Van Eyken E, Stephan DA, et al (2010) A genome-wide association study for age-related hearing impairment in the Saami. Eur J Hum Genet In press. 10.1038/ejhg.2009.234 PMC2987344 20068591

[7] Friedman RA, Van Laer L, Huentelman MJ, Sheth SS, Van Eyken E, et al (2009) GRM7 variants confer susceptibility to age-related hearing impairment. Human Molecular Genetics 18: 785–796. 19047183 10.1093/hmg/ddn402 PMC2638831

[8] Ter HG, Venker-van Haagen AJ, van den Brom WE, van Sluijs FJ, Smoorenburg GF (2008) Effects of aging on brainstem responses to toneburst auditory stimuli: a cross-sectional and longitudinal study in dogs. J Vet Intern Med 22: 937–945. 18564227 10.1111/j.1939-1676.2008.0126.x

[9] Knowles K, Blauch B, Leipold H, Cash W, Hewett J (1989) Reduction of spiral ganglion neurons in the aging canine with hearing loss. Zentralbl Veterinarmed A 36: 188–199. 2499997 10.1111/j.1439-0442.1989.tb00719.x

[10] Shimada A, Ebisu M, Morita T, Takeuchi T, Umemura T (1998) Age-Related Changes in the Cochlea and Cochlear Nuclei of Dogs. The Journal of Veterinary Medical Science 60: 41–48. 9492359 10.1292/jvms.60.41

[11] Schuknecht HF, Gacek MR (1993) Cochlear pathology in presbycusis. Ann Otol Rhinol Laryngol 102: 1–16. 10.1177/00034894931020S101 8420477

[12] Ter HG, de Groot JC, Venker-van Haagen AJ, van Sluijs FJ, Smoorenburg GF (2009) Effects of aging on inner ear morphology in dogs in relation to brainstem responses to toneburst auditory stimuli. J Vet Intern Med 23: 536–543. 19645839 10.1111/j.1939-1676.2009.0290.x

[13] United States Border Collie Handlers' Association 2011 Sheep Dog Pedigrees. Available: www.usbcha.com/events/sheep/pedigrees.html. Accessed 2012 May 23.

[14] Strain GM (1996) Aetiology, prevalence and diagnosis of deafness in dogs and cats. Br Vet J 152: 17–36. 8634862 10.1016/s0007-1935(96)80083-2

[15] Shworak NW, Liu J, Petros LM, Zhang L, Kobayashi M, et al (1999) Multiple isoforms of heparan sulfate D-glucosaminyl 3-O-sulfotransferase. Isolation, characterization, and expression of human cdnas and identification of distinct genomic loci. J Biol Chem 274: 5170–5184. 9988767 10.1074/jbc.274.8.5170

[16] Zwaenepoel I, Mustapha M, Leibovici M, Verpy E, Goodyear R, et al (2002) Otoancorin, an inner ear protein restricted to the interface between the apical surface of sensory epithelia and their overlying acellular gels, is defective in autosomal recessive deafness DFNB22. Proceedings of the National Academy of Sciences of the United States of America 99: 6240–6245. 11972037 10.1073/pnas.082515999 PMC122933

[17] Rowe TM, Rizzi M, Hirose K, Peters GA, Sen GC (2006) A role of the double-stranded RNA-binding protein PACT in mouse ear development and hearing. Proc Natl Acad Sci U S A 103: 5823–5828 0601287103 [pii];10.1073/pnas.0601287103 [doi]. 16571658 10.1073/pnas.0601287103 PMC1458657

[18] Lockhart PJ, Hulihan M, Lincoln S, Hussey J, Skipper L, et al (2004) Identification of the human ubiquitin specific protease 31 (USP31) gene: structure, sequence and expression analysis. DNA Seq 15: 9–14. 15354349 10.1080/10855660310001638197

[19] Lang H, Schulte BA, Zhou D, Smythe N, Spicer SS, et al (2006) Nuclear factor kappaB deficiency is associated with auditory nerve degeneration and increased noise-induced hearing loss. J Neurosci 26: 3541–3550 26/13/3541 [pii];10.1523/JNEUROSCI.2488-05.2006 [doi]. 16571762 10.1523/JNEUROSCI.2488-05.2006 PMC2897814

[20] Tzimas C, Michailidou G, Arsenakis M, Kieff E, Mosialos G, et al (2006) Human ubiquitin specific protease 31 is a deubiquitinating enzyme implicated in activation of nuclear factor-kappaB. Cell Signal 18: 83–92 S0898-6568(05)00074-4 [pii];10.1016/j.cellsig.2005.03.017 [doi]. 16214042 10.1016/j.cellsig.2005.03.017

[21] Kappo MA, Eiso AB, Hassem F, Atkinson RA, Faro A, et al (2012) Solution Structure of RING Finger-like Domain of Retinoblastoma-binding Protein-6 (RBBP6) Suggests It Functions as a U-box. J Biol Chem 287 (10) 7146–58. 22130672 10.1074/jbc.M110.217059 PMC3293548

[22] Alvarez CE, Akey JM (2012) Copy number variation in the domestic dog. Mamm Genome 23: 144–163 10.1007/s00335-011-9369-8 [doi]. 22138850 10.1007/s00335-011-9369-8

[23] Purcell S, Neale B, Todd-Brown K, Thomas L, Ferreira MA, et al (2007) PLINK: a tool set for whole-genome association and population-based linkage analyses. Am J Hum Genet 81: 559–575. 17701901 10.1086/519795 PMC1950838

[24] Kang HM, Sul JH, Service SK, Zaitlen NA, Kong Sy, et al (2010) Variance component model to account for sample structure in genome-wide association studies. Nat Genet 42: 348–354. 20208533 10.1038/ng.548 PMC3092069

[25] Langmead B, Trapnell C, Pop M, Salzberg SL (2009) Ultrafast and memory-efficient alignment of short DNA sequences to the human genome. Genome Biol 10: R25 gb-2009-10-3-r25 [pii];10.1186/gb-2009-10-3-r25 [doi]. 19261174 10.1186/gb-2009-10-3-r25 PMC2690996

[26] Li H, Handsaker B, Wysoker A, Fennell T, Ruan J, et al (2009) The Sequence Alignment/Map format and SAMtools. Bioinformatics 25: 2078–2079 btp352 [pii];10.1093/bioinformatics/btp352 [doi]. 19505943 10.1093/bioinformatics/btp352 PMC2723002

[27] Quinlan AR, Hall IM (2010) BEDTools: a flexible suite of utilities for comparing genomic features. Bioinformatics 26: 841–842 btq033 [pii];10.1093/bioinformatics/btq033 [doi]. 20110278 10.1093/bioinformatics/btq033 PMC2832824

[28] McKenna A, Hanna M, Banks E, Sivachenko A, Cibulskis K, et al (2010) The Genome Analysis Toolkit: a MapReduce framework for analyzing next-generation DNA sequencing data. Genome Res 20: 1297–1303 gr.107524.110 [pii];10.1101/gr.107524.110 [doi]. 20644199 10.1101/gr.107524.110 PMC2928508

[29] Albers CA, Lunter G, MacArthur DG, McVean G, Ouwehand WH, et al (2011) Dindel: accurate indel calls from short-read data. Genome Res 21: 961–973 gr.112326.110 [pii];10.1101/gr.112326.110 [doi]. 20980555 10.1101/gr.112326.110 PMC3106329

[30] Wang K, Li M, Hakonarson H (2010) ANNOVAR: functional annotation of genetic variants from high-throughput sequencing data. Nucl Acids Res 38: e164. 20601685 10.1093/nar/gkq603 PMC2938201

[31] Kent WJ, Sugnet CW, Furey TS, Roskin KM, Pringle TH, et al (2002) The Human Genome Browser at UCSC. Genome Res 12: 996–1006. 12045153 10.1101/gr.229102 PMC186604

[32] Willer CJ, Li Y, Abecasis GR (2010) METAL: fast and efficient meta-analysis of genomewide association scans. Bioinformatics 26: 2190–2191 btq340 [pii];10.1093/bioinformatics/btq340 [doi]. 20616382 10.1093/bioinformatics/btq340 PMC2922887

[33] Chen ZQ, Annilo T, Shulenin S, Dean M (2004) Three ATP-binding cassette transporter genes, Abca14, Abca15, and Abca16, form a cluster on mouse chromosome 7F3. Mamm Genome 15: 335–43. 15170222 10.1007/s00335-004-2281-8

