# Supplementary figures and images for: Variation in Genes Related to Cochlear Biology Is Strongly Associated with Adult-Onset Deafness in Border Collies
Source: PLoS Genet. 2012 Sep 13;8(9):e1002898. doi: 10.1371/journal.pgen.1002898 (PMC3441646; doi:10.1371/journal.pgen.1002898)

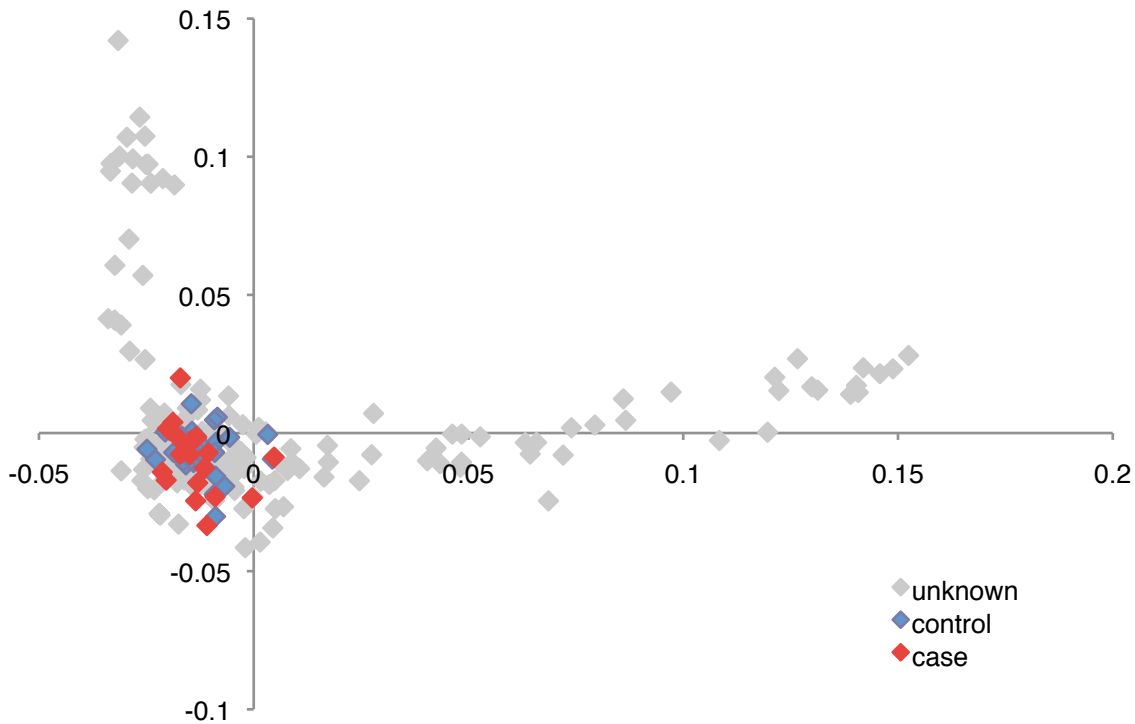

Supplement: Figure S1 — Multi-dimensional scaling (MDS) vector plots of Border Collies used for deafness analysis. MDS1 x MDS2 based on data from all unrelated Border Collies genotyped by our group for ongoing studies of behavior. A total of 10 MDS covariates were calculated for all Border Collies using a subset of unlinked (r2<0.8) whole genome SNP data (∼22 k markers total) in PLINK. Matched controls (blue) were selected based on genetic similarity to cases (red) for the primary GWAS. Samples in gray are shown to demonstrate the overall genetic diversity found in our entire Border Collie cohort. (PDF) [file pgen.1002898.s001.pdf]

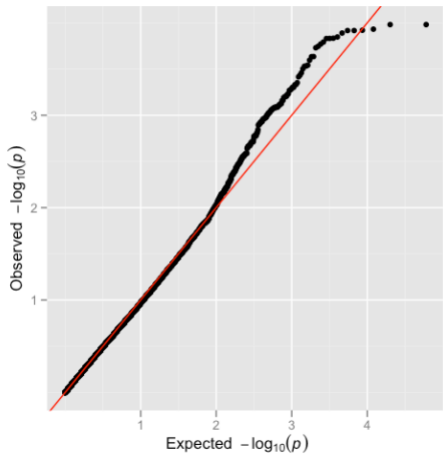

Supplement: Figure S2 — Q-Q plot of GWAS analysis for adult-onset deafness in Border Collies. Expected versus observed –log10(p-value) for the primary GWAS are plotted for each marker; the red line indicates the null distribution. Given the strong association signal by multiple linked markers on CFA6, findings with p-values less than 0.0001 were removed from this plot to avoid skewing the graphical distribution at high observed p-values. The Q-Q plot for this analysis suggests that there is minimal population stratification in this sample, as the majority of points lie on the null distribution. (PDF) [file pgen.1002898.s002.pdf]

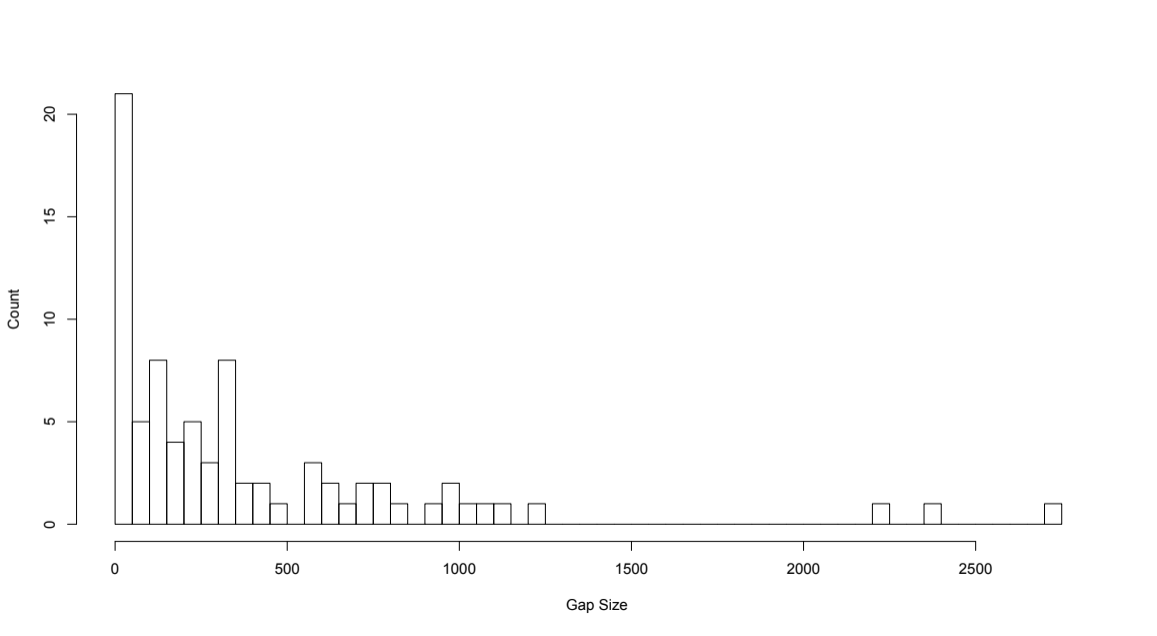

Supplement: Figure S3 — Number of gaps in the canFam2 assembly from 23 Mb to 29 Mb by size. There were 80 gaps in the canFam2 assembly ranging from 1 bp to 2707 bp (mean = 388 bp; median = 217 bp). The gaps sum to 31089 bp, or about 0.5% of the sequence within the region. (PDF) [file pgen.1002898.s003.pdf]

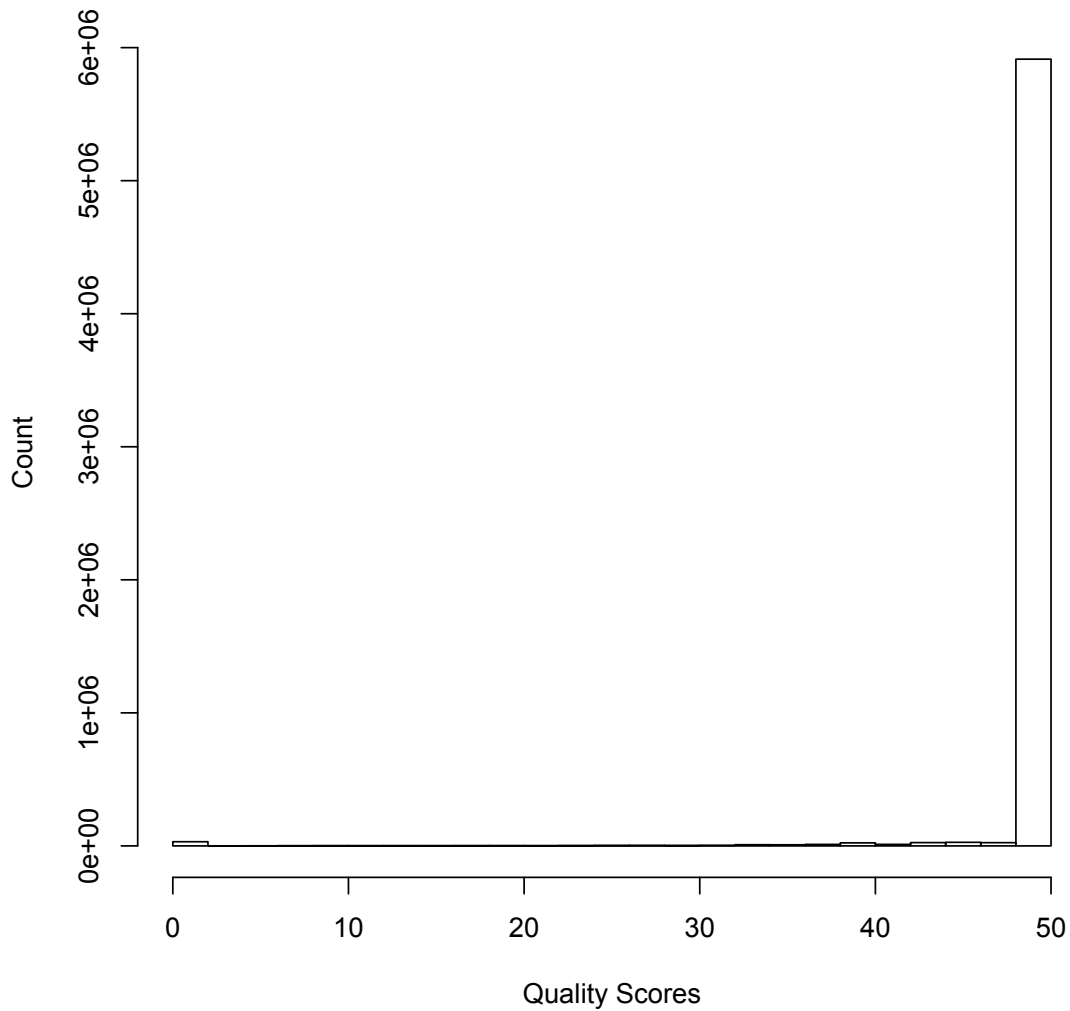

Supplement: Figure S4 — Sequencing assembly quality scores of target capture region in the canFam2 assembly. As shown, most (98%) of the bases in the assembly have quality scores equal or bigger than 40 (deemed high confidence). (PDF) [file pgen.1002898.s004.pdf]
