# Supplementary material for: Variation in Genes Related to Cochlear Biology Is Strongly Associated with Adult-Onset Deafness in Border Collies
Source: PLoS Genet. 2012 Sep 13;8(9):e1002898. doi: 10.1371/journal.pgen.1002898 (PMC3441646; doi:10.1371/journal.pgen.1002898)
Supplement: Table S1 — Sample summary. Samples for the primary genome-wide association study (GWAS) and targeted genotyping were collected from two countries, with breakdown of cases and controls provided for a total of 405 Border Collies. (DOCX) [file pgen.1002898.s005.docx]

| **Table S1: Sample summary.** | | | | |
| --- | --- | --- | --- | --- |
| **Sample** | **Country of Origin** | **Cases** | **Controls** | **Total** |
| Primary GWAS | USA | 20 | 28 | 48 |
| Follow-up genotyping | USA | 0 | 14 | 14 |
|  | Finland | 3 | 59 | 62 |
| Replication | USA | 16 | 265 | 281 |
| **Totals** |  | 39 | 366 | 405 |
