# Supplementary material for: Variation in Genes Related to Cochlear Biology Is Strongly Associated with Adult-Onset Deafness in Border Collies
Source: PLoS Genet. 2012 Sep 13;8(9):e1002898. doi: 10.1371/journal.pgen.1002898 (PMC3441646; doi:10.1371/journal.pgen.1002898)
Supplement: Table S2 — Next-generation sequencing statistics. Each sample was run in a single lane for 76 sequencing cycles. Given the high number of variants called, we first filtered variants with regard to their genotype in cases and controls, filtering for variants called homozygous in the case sample and called not homozygous for that variant in either of the controls. We then focused on exonic and potentially functional non-coding variants, with priority given to top biological candidates. For a summary of SNPs as annotated in ANNOVAR, see Table S4. (DOCX) [file pgen.1002898.s006.docx]

| **Table S2: Next generation sequencing statistics.** | | | |
| --- | --- | --- | --- |
|  | **Control 1** | **Control 2** | **Case** |
| Total reads | 36,270,529 | 30,867,026 | 32,404,825 |
| Aligned reads | 33,564,330 | 27,899,663 | 30,252,775 |
| %Aligned reads | 92.5% | 90.4% | 93.4% |
|  |  |  |  |
| Mean bait coverage (X) | 905.9 | 658.2 | 785.3 |
| Mean target coverage (X) | 548.2 | 403.7 | 483.7 |
| Fold enrichment | 868.3 | 759.0 | 835.2 |
|  |  |  |  |
| %Target >2X | 77.6% | 78.7% | 81.6% |
| %Target >10X | 75.7% | 73.1% | 77.1% |
| %Target >20X | 73.1% | 68.6% | 73.8% |
| %Target >30X | 71.1% | 65.2% | 71.1% |
|  |  |  |  |
| Reads on target | 26,286,959 | 19,356,053 | 23,193,286 |
| % Aligned reads on target | 78% | 69% | 77% |
