# Supplementary material for: Variation in Genes Related to Cochlear Biology Is Strongly Associated with Adult-Onset Deafness in Border Collies
Source: PLoS Genet. 2012 Sep 13;8(9):e1002898. doi: 10.1371/journal.pgen.1002898 (PMC3441646; doi:10.1371/journal.pgen.1002898)
Supplement: Table S3 — Summary of variants homozygous in case and not in controls using ANNOVAR. Number of SNPs of different locations and functional relevance as annotated by ANNOVAR are provided for total experiment and for which the variants are homozygous in the case sample but not in either control sample (assuming a recessive mode of inheritance as suggested by homozygous risk haplotypes observed in GWAS cases). (DOCX) [file pgen.1002898.s007.docx]

| **Table S3: Summary of variants homozygous in case and not in controls using ANNOVAR.** | | |
| --- | --- | --- |
|  | **In target** | **In target & homozygous in case**  **but not in controls** |
| Downstream | 86 | 23 |
| Exonic | 106 | 26 |
| Synonymous | 67 | 19 |
| Nonsynonymous | 38 | 7 |
| Stopgain | 1 | 0 |
| Intergenic | 953 | 140 |
| Intronic | 3538 | 718 |
| Splicing | 2 | 0 |
| Upstream | 96 | 17 |
| 3' UTR | 10 | 2 |
| 5' UTR | 28 | 9 |
| *3’ UTR: untranslated 3’; 5’ UTR: untranslated 5’* | | |
