# Supplementary material for: Variation in Genes Related to Cochlear Biology Is Strongly Associated with Adult-Onset Deafness in Border Collies
Source: PLoS Genet. 2012 Sep 13;8(9):e1002898. doi: 10.1371/journal.pgen.1002898 (PMC3441646; doi:10.1371/journal.pgen.1002898)
Supplement: Table S4 — Exonic variants for deafness on CFA6. A list of the 26 exonic variants for CFA6 plus annotations is given in Table S4. Gene annotations and predicted amino acid (AA) changes (single letter AA abbreviations flanking AA position) are given with reference to the gene in the human unless the gene is not present in human, in which case it is given for the species noted (Mus – mouse, Sac – yeast, Bos – cow, Rat – rat). Non-synonymous SNPs (nsSNP) are marked in bold. In addition to the called genotypes for each sample, the sequence coverage for that SNP is also provided. Finally, the phastCons4Way score provides a measure of conservation for each position, where values closer to 1 indicate the base is more highly conserved across species. Conservation is based on alignment with human (hg17), mouse (mm6), and rat (rn3). CFA: canine chromosome; Position: base position; Ref: reference allele from genome; Alt: alternate allele observed in sample[s]; genotype: 0 = reference allele, 1 = alternate allele; phastCons score: phastCons4Way score from UCSC genome browser. Of the 26 putative exonic variants, only 8 were annotated to be non-synonymous changes. Four nsSNPs were found in Abca14, which was the gene with the most nsSNPs. Abca14 is an ATP binding cassette transporter gene that has only been annotated in the genomes of rodents [33]. Conservation scores for all four of these nsSNPs were low, suggesting that this gene may not be active and thus tolerant of non-synonymous changes more readily. There was an additional gene containing an nsSNPs, that is not readily linked to hearing function or expression (EEF2K). (DOCX) [file pgen.1002898.s008.docx]

| **Table S4: Exonic variants for deafness on CFA6.** | | | | | | | | | | | | |
| --- | --- | --- | --- | --- | --- | --- | --- | --- | --- | --- | --- | --- |
| **Type** | **Locus** | **CFA** | **Position** | **Ref** | **Alt** | **Control 1** | | **Control 2** | | **Case** | | **phastCons Score** |
|  |  |  |  |  |  | **Genotype** | **Coverage** | **Genotype** | **Coverage** | **Genotype** | **Coverage** |  |
| **nsSNP** | ***RBBP6*, exon18, p.T1397N** | **6** | **24500625** | **G** | **T** | **0/0** | **249** | **0/0** | **72** | **1/1** | **226** | **0.001** |
| ssSNP | *RBBP6*, exon11, p.E445E | 6 | 24508479 | T | C | 1/1 | 248 | 1/1 | 123 | 0/0 | 226 | 0.925 |
| ssSNP | *ERN2*, exon9, p.L260L | 6 | 25178743 | T | C | 0/0 | 236 | 0/1 | 241 | 1/1 | 240 | 0.871 |
| ssSNP | *ERN2*, exon19, p.F578F | 6 | 25185894 | C | T | 1/1 | 233 | 0/1 | 223 | 0/0 | 214 | 0.925 |
| ssSNP | *ERN2*, exon25, p.D838D | 6 | 25188304 | C | T | 1/1 | 208 | 0/1 | 245 | 0/0 | 244 | 0.949 |
| ssSNP | *PLK1*, exon9, p.L511L | 6 | 25189840 | A | G | 1/1 | 233 | 0/1 | 236 | 0/0 | 244 | 0.792 |
| ssSNP | *PLK1*, exon9, p.E488E | 6 | 25189907 | T | C | 1/1 | 242 | 0/1 | 247 | 0/0 | 249 | 0.831 |
| ssSNP | *PLK1*, exon1, p.K97K | 6 | 25199818 | C | T | 1/1 | 238 | 0/1 | 233 | 0/0 | 241 | 0.971 |
| **nsSNP** | ***USP31*, exon17, p.I847V** | **6** | **25714052** | **A** | **G** | **0/0** | **245** | **0/1** | **246** | **1/1** | **245** | **0.950** |
| **nsSNP** | ***EEF2K*, exon1, p.N62K** | **6** | **26442657** | **G** | **T** | **1/1** | **249** | **0/1** | **250** | **0/0** | **249** | **0.627** |
| **nsSNP** | ***Abca14* (Mus), exon26, p.M1292L** | **6** | **26909869** | **T** | **G** | **0/0** | **53** | **0/1** | **18** | **1/1** | **16** | **0.058** |
| **nsSNP** | ***Abca14* (Mus), exon23, p.L1134I** | **6** | **26924547** | **G** | **T** | **0/0** | **230** | **0/1** | **63** | **1/1** | **189** | **0.013** |
| **nsSNP** | ***Abca14* (Mus), exon16, p.V699I** | **6** | **26951574** | **C** | **T** | **0/0** | **243** | **0/1** | **247** | **1/1** | **247** | **0.045** |
| **nsSNP** | ***Abca14* (Mus), exon9, p.I472M** | **6** | **26972673** | **T** | **C** | **0/0** | **220** | **0/1** | **114** | **1/1** | **242** | **0.145** |
| ssSNP | *DNAH3*, exon56, p.R2825R | 6 | 27436889 | G | A | 0/0 | 247 | 0/1 | 246 | 1/1 | 246 | 0.980 |
| ssSNP | *DNAH3*, exon60, p.D3733D | 6 | 27456701 | C | T | 0/0 | 243 | 0/1 | 243 | 1/1 | 245 | 0.031 |
| ssSNP | *LOC57020*, exon11, p.D462D | 6 | 27898482 | G | A | 0/0 | 244 | 0/0 | 246 | 1/1 | 242 | 0.352 |
| ssSNP | *C16orf62*, exon26, p.T707T | 6 | 28661889 | C | T | 0/0 | 249 | 0/0 | 67 | 1/1 | 245 | 0.972 |
| ssSNP | *C16orf62*, exon9, p.S232S | 6 | 28709547 | A | G | 0/0 | 243 | 0/0 | 226 | 1/1 | 246 | 0.972 |
| ssSNP | *CP110*, exon3, p.K201K | 6 | 28750373 | C | T | 0/0 | 191 | 0/0 | 91 | 1/1 | 221 | 0.463 |
| ssSNP | *CP110*, exon1, p.L142L | 6 | 28753519 | T | C | 1/1 | 248 | 0/1 | 148 | 0/0 | 230 | 0.918 |
| ssSNP | *CP110*, exon1, p.T17T | 6 | 28753894 | G | A | 0/0 | 247 | 0/0 | 222 | 1/1 | 246 | 0.518 |
| ssSNP | *COQ7* (Sac), exon4, p.K131K | 6 | 28948448 | A | G | 0/0 | 240 | 0/0 | 249 | 1/1 | 249 | 0.923 |
| ssSNP | *ITPRIPL2*, exon1, p.A365A | 6 | 28978457 | C | T | 0/0 | 55 | 0/0 | 149 | 1/1 | 88 | 0.880 |
| ssSNP | *ITPRIPL2*, exon1, p.L414L | 6 | 28978602 | T | C | 0/0 | 80 | 0/0 | 165 | 1/1 | 102 | 0.141 |
| ssSNP | *ITPRIPL2*, exon1, p.L415L | 6 | 28978605 | T | C | 0/0 | 77 | 0/0 | 156 | 1/1 | 104 | 0.063 |
