# Supplementary material for: Variation in Genes Related to Cochlear Biology Is Strongly Associated with Adult-Onset Deafness in Border Collies
Source: PLoS Genet. 2012 Sep 13;8(9):e1002898. doi: 10.1371/journal.pgen.1002898 (PMC3441646; doi:10.1371/journal.pgen.1002898)
Supplement: Table S5 — List of primers used in dye-terminator sequencing. Follow-up sequencing was performed in additional samples for three variants. The PCR conditions used are described in Materials and Methods . (DOCX) [file pgen.1002898.s009.docx]

| **Table S5: List of primers used in dye-terminator sequencing.** | | |
| --- | --- | --- |
| **SNP** | **Forward** | **Reverse** |
| Chr6.24500625 | 5’-TGAGGGACTGGAACTGCTCT-3’ | 5’-AGTCCTGTGCGGAAATCTGA-3’ |
| Chr6.25681850 | 5’-TTTTGTTTGGCTGCCTTCTC-3’ | 5’-TGCCCACAGAAAAATCCCTA-3’ |
| Chr6.25714052 | 5’-GCCTTCCTCCCTTCTTCAGT-3’ | 5’-CGAAGGAGATGACACGGAGT-3’ |
