# Supplementary material for: Variation in Genes Related to Cochlear Biology Is Strongly Associated with Adult-Onset Deafness in Border Collies
Source: PLoS Genet. 2012 Sep 13;8(9):e1002898. doi: 10.1371/journal.pgen.1002898 (PMC3441646; doi:10.1371/journal.pgen.1002898)
Supplement: Table S6 — List of predicted genes targeted for target capture sequencing and probe coverage by gene. Position of targeted region in canFam2 and number of targets per gene are listed for all target capture regions. An “n/a” is used when a target gene is within another predicted gene that has already been targeted. (DOCX) [file pgen.1002898.s010.docx]

| **Table S6: List of predicted genes targeted for target capture sequencing and probe coverage by gene.** | | | | |
| --- | --- | --- | --- | --- |
| **Gene ID** | **Gene name** | **Targeted region** | **No. of target regions within gene** |  |
| NM_006040 | *HS3ST4* | chr6:23237568-23638888 | 24 |  |
| NM_001012981 | *ZKSCAN2* | chr6:23976533-23997352 | 25 |  |
| NM_001169 | *AQP8* | chr6:24003232-24012663 | 9 |  |
| NM_016309 | *LCMT1* | chr6:24046309-24086693 | 48 |  |
| NM_001076019 | *C8H9orf30* | chr6:24057127-24060285 | n/a |  |
| NM_001016739 | *c9orf30* | chr6:24057154-24059998 | n/a |  |
| NM_018054 | *ARHGAP17* | chr6:24099958-24196585 | 101 |  |
| NM_052944 | *SLC5A11* | chr6:24201588-24237597 | 45 |  |
| NM_014494 | *TNRC6A* | chr6:24265253-24373846 | 114 |  |
| NM_006910 | *RBBP6* | chr6:24498008-24531951 | 30 |  |
| NM_006539 | *CACNG3* | chr6:24644639-24732130 | 97 |  |
| NM_002738 | *PRKCB* | chr6:24760568-25086234 | 389 |  |
| NM_022097 | *CHP2* | chr6:25148807-25153168 | 4 |  |
| NM_033266 | *ERN2* | chr6:25174871-25190051 | 12 |  |
| NM_005030 | *PLK1* | chr6:25188004-25201121 | 11 |  |
| NM_032486 | *DCTN5* | chr6:25208311-25234185 | 26 |  |
| NM_024675 | *PALB2* | chr6:25232485-25262468 | 31 |  |
| NM_005003 | *NDUFAB1* | chr6:25265061-25276929 | 18 |  |
| NM_019116 | *UBFD1* | chr6:25282922-25300748 | 12 |  |
| NR_003501 | *EARS2* | chr6:25298942-25322502 | 30 |  |
| NM_015044 | *GGA2* | chr6:25340027-25369230 | 24 |  |
| NM_153603 | *COG7* | chr6:25378018-25460052 | 73 |  |
| NM_000336 | *SCNN1B* | chr6:25462920-25487946 | 20 |  |
| NM_001039 | *SCNN1G* | chr6:25593643-25618204 | 27 |  |
| NM_020718 | *USP31* | chr6:25649946-25722762 | 78 |  |
| NM_006043 | *HS3ST2* | chr6:25842382-25935014 | 107 |  |
| NM_144672 | *OTOA* | chr6:26134411-26197802 | 78 |  |
| NM_001077180 | *METTL9* | chr6:26208563-26245138 | 33 |  |
| NM_030691 | *Igsf6* | chr6:26216896-26223970 | n/a |  |
| NM_001802 | *CDR2* | chr6:26324472-26345913 | 19 |  |
| NM_018119 | *POLR3E* | chr6:26356857-26387924 | 29 |  |
| NM_013302 | *EEF2K* | chr6:26396174-26463173 | 69 |  |
| NM_173615 | *VWA3A* | chr6:26500885-26562226 | 58 |  |
| NM_001164579 | *C16orf52* | chr6:26568920-26637167 | 71 |  |
| NM_173806 | *PDZD9* | chr6:26642205-26656344 | 13 |  |
| NM_003366 | *UQCRC2* | chr6:26655187-26682978 | 24 |  |
| NM_026458 | *Abca14* | chr6:26703127-26835863 | 63 |  |
| NR_024051 | *Abca16* | chr6:26887834-27195268 | 155 |  |
| NM_001888 | *CRYM* | chr6:27213760-27232848 | 21 |  |
| NM_145865 | *ANKS4B* | chr6:27236043-27246352 | 9 |  |
| NM_003460 | *ZP2* | chr6:27256838-27269770 | 13 |  |
| NM_020422 | *TMEM159* | chr6:27282092-27294013 | 13 |  |
| NM_017539 | *DNAH3* | chr6:27304543-27466095 | 157 |  |
| NM_020424 | *LYRM1* | chr6:27467260-27489430 | 20 |  |
| NM_173475 | *DCUN1D3* | chr6:27487425-27525621 | 32 |  |
| NM_030941 | *LOC81691* | chr6:27532246-27578425 | 35 |  |
| NM_001142725 | *ERI2* | chr6:27576410-27587378 | 8 |  |
| NM_005622 | *ACSM3* | chr6:27584660-27605071 | 20 |  |
| NM_017736 | *THUMPD1* | chr6:27633650-27644499 | 8 |  |
| NM_178414 | *Acsm4* | chr6:27643513-27671891 | 33 |  |
| NM_001087266 | *nono* | chr6:27683924-27687135 | 1 |  |
| NR_003277 | *LOC728643* | chr6:27687755-27690305 | 2 |  |
| NM_001101952 | *ACSM2A* | chr6:27892180-27921107 | 22 |  |
| NM_017888 | *ACSM5* | chr6:27934924-27967023 | 24 |  |
| NM_174924 | *PDILT* | chr6:27970602-28014520 | 45 |  |
| NM_003361 | *UMOD* | chr6:28018265-28035478 | 22 |  |
| NM_001502 | *GP2* | chr6:28038975-28055408 | 21 |  |
| NM_001002911 | *GPR139* | chr6:28276437-28318406 | 48 |  |
| NM_016235 | *GPRC5B* | chr6:28439878-28462832 | 24 |  |
| NM_153208 | *IQCK* | chr6:28462315-28593455 | 140 |  |
| NM_001012991 | *C16orf88* | chr6:28594515-28609132 | 12 |  |
| NM_020314 | *C16orf62* | chr6:28621208-28731692 | 130 |  |
| NM_014711 | *CP110* | chr6:28739342-28767554 | 21 |  |
| NM_016641 | *GDE1* | chr6:28767489-28787280 | 23 |  |
| NM_001105248 | *TMC5* | chr6:28792188-28836777 | 49 |  |
| NM_001160364 | *TMC7* | chr6:28898390-28938484 | 52 |  |
| NM_016138 | *COQ7* | chr6:28939812-28950941 | 9 |  |
| NM_001033380 | *Itpripl2* | chr6:28975917-28984544 | 9 |  |
| NM_016524 | *SYT17* | chr6:29031381-29103760 | 82 |  |
| NR_024436 | *LOC728276* | chr6:29109495-29127642 | 18 |  |
| NM_015092 | *SMG1* | chr6:29257813-29337314 | 74 |  |
| NM_015161 | *ARL6IP1* | chr6:29337969-29348302 | 9 |  |
| NM_001019 | *RPS15A* | chr6:29351515-29360443 | 5 |  |
