# Supplementary material for: Variation in Genes Related to Cochlear Biology Is Strongly Associated with Adult-Onset Deafness in Border Collies
Source: PLoS Genet. 2012 Sep 13;8(9):e1002898. doi: 10.1371/journal.pgen.1002898 (PMC3441646; doi:10.1371/journal.pgen.1002898)
Supplement: Table S7 — Gene coverage information by gene for each target capture sample. Percent bases covered and average coverage depth is provided for each sample that was sequenced using next-generation technology. Coverage is listed by target gene. (DOCX) [file pgen.1002898.s011.docx]

| **Table S7: Gene coverage information by gene for each target capture sample.** | | | | | | | | | | | |
| --- | --- | --- | --- | --- | --- | --- | --- | --- | --- | --- | --- |
| **Chr** | **Start** | **End** | **Gene** | **%Bases >1X in any sample** |  | **%Bases >1X in Control 1** | **Control 1 Avg Coverage (X)** | **%Bases >1X in Control 2** | **Control 2 Avg Coverage (X)** | **%Bases >1X in Case** | **Case Avg Coverage (X)** |
| 6 | 23237568 | 23637888 | *HS3ST4* | 13% |  | 5% | 37 | 8% | 36 | 9% | 40 |
| 6 | 23977533 | 23996352 | *ZKSCAN2* | 100% |  | 97% | 728 | 99% | 539 | 100% | 691 |
| 6 | 24004232 | 24011663 | *AQP8* | 95% |  | 87% | 306 | 91% | 439 | 94% | 377 |
| 6 | 24047309 | 24085693 | *LCMT1* | 89% |  | 81% | 660 | 84% | 557 | 87% | 587 |
| 6 | 24058127 | 24059285 | *C8H9orf30* | 100% |  | 100% | 914 | 100% | 879 | 100% | 988 |
| 6 | 24058154 | 24058998 | *c9orf30* | 100% |  | 100% | 938 | 100% | 1008 | 100% | 1103 |
| 6 | 24100958 | 24195585 | *ARHGAP17* | 92% |  | 87% | 761 | 89% | 666 | 91% | 747 |
| 6 | 24202588 | 24236597 | *SLC5A11* | 92% |  | 82% | 617 | 87% | 529 | 90% | 613 |
| 6 | 24266253 | 24372846 | *TNRC6A* | 95% |  | 90% | 750 | 90% | 468 | 93% | 621 |
| 6 | 24499008 | 24530951 | *RBBP6* | 99% |  | 96% | 550 | 96% | 140 | 98% | 262 |
| 6 | 24645639 | 24731130 | *CACNG3* | 92% |  | 85% | 557 | 88% | 498 | 90% | 558 |
| 6 | 24761568 | 25085234 | *PRKCB* | 91% |  | 84% | 634 | 87% | 549 | 90% | 629 |
| 6 | 25149807 | 25152168 | *CHP2* | 100% |  | 100% | 1399 | 100% | 1391 | 100% | 1598 |
| 6 | 25175871 | 25189051 | *ERN2* | 91% |  | 86% | 654 | 90% | 853 | 90% | 720 |
| 6 | 25189004 | 25200121 | *PLK1* | 93% |  | 87% | 522 | 90% | 683 | 91% | 629 |
| 6 | 25209311 | 25233185 | *DCTN5* | 85% |  | 76% | 465 | 81% | 263 | 84% | 425 |
| 6 | 25233485 | 25261468 | *PALB2* | 91% |  | 80% | 477 | 81% | 238 | 87% | 346 |
| 6 | 25266061 | 25275929 | *NDUFAB1* | 99% |  | 94% | 773 | 97% | 570 | 98% | 737 |
| 6 | 25283922 | 25299748 | *UBFD1* | 97% |  | 92% | 618 | 96% | 614 | 97% | 643 |
| 6 | 25299942 | 25321502 | *EARS2* | 94% |  | 86% | 582 | 91% | 606 | 93% | 588 |
| 6 | 25341027 | 25368230 | *GGA2* | 88% |  | 83% | 821 | 83% | 668 | 86% | 736 |
| 6 | 25379018 | 25459052 | *COG7* | 83% |  | 75% | 596 | 75% | 500 | 80% | 582 |
| 6 | 25463920 | 25486946 | *SCNN1B* | 89% |  | 79% | 437 | 86% | 562 | 87% | 446 |
| 6 | 25594643 | 25617204 | *SCNN1G* | 97% |  | 87% | 693 | 94% | 673 | 95% | 713 |
| 6 | 25650946 | 25721762 | *USP31* | 94% |  | 90% | 761 | 90% | 407 | 93% | 556 |
| 6 | 25843382 | 25934014 | *HS3ST2* | 93% |  | 86% | 573 | 89% | 586 | 91% | 601 |
| 6 | 26135411 | 26196802 | *OTOA* | 91% |  | 82% | 544 | 87% | 424 | 88% | 496 |
| 6 | 26209563 | 26244138 | *METTL9* | 93% |  | 88% | 496 | 87% | 208 | 92% | 336 |
| 6 | 26217896 | 26222970 | *Igsf6* | 100% |  | 100% | 708 | 97% | 322 | 100% | 386 |
| 6 | 26325472 | 26344913 | *CDR2* | 88% |  | 79% | 350 | 82% | 168 | 86% | 278 |
| 6 | 26357857 | 26386924 | *POLR3E* | 93% |  | 86% | 657 | 91% | 842 | 91% | 743 |
| 6 | 26397174 | 26462173 | *EEF2K* | 87% |  | 74% | 436 | 82% | 449 | 84% | 498 |
| 6 | 26501885 | 26561226 | *VWA3A* | 90% |  | 80% | 623 | 84% | 569 | 89% | 647 |
| 6 | 26569920 | 26636167 | *C16orf52* | 83% |  | 76% | 273 | 74% | 70 | 79% | 145 |
| 6 | 26643205 | 26655344 | *PDZD9* | 99% |  | 95% | 721 | 95% | 475 | 99% | 616 |
| 6 | 26656187 | 26681978 | *UQCRC2* | 90% |  | 85% | 591 | 83% | 286 | 86% | 453 |
| 6 | 26704127 | 26834863 | *Abca14* | 43% |  | 37% | 135 | 35% | 34 | 37% | 57 |
| 6 | 26888834 | 27194268 | *Abca16* | 45% |  | 38% | 165 | 38% | 46 | 41% | 77 |
| 6 | 27214760 | 27231848 | *CRYM* | 99% |  | 96% | 680 | 98% | 471 | 99% | 621 |
| 6 | 27237043 | 27245352 | *ANKS4B* | 95% |  | 92% | 828 | 90% | 434 | 91% | 618 |
| 6 | 27257838 | 27268770 | *ZP2* | 100% |  | 100% | 1154 | 99% | 495 | 100% | 871 |
| 6 | 27283092 | 27293013 | *TMEM159* | 92% |  | 89% | 663 | 86% | 384 | 89% | 516 |
| 6 | 27305543 | 27465095 | *DNAH3* | 84% |  | 77% | 560 | 77% | 336 | 81% | 456 |
| 6 | 27468260 | 27488430 | *LYRM1* | 99% |  | 93% | 710 | 96% | 441 | 97% | 554 |
| 6 | 27488425 | 27524621 | *DCUN1D3* | 97% |  | 93% | 709 | 95% | 430 | 96% | 581 |
| 6 | 27533246 | 27577425 | *LOC81691* | 69% |  | 65% | 312 | 64% | 144 | 67% | 229 |
| 6 | 27577410 | 27586378 | *ERI2* | 100% |  | 95% | 416 | 93% | 88 | 99% | 163 |
| 6 | 27585660 | 27604071 | *ACSM3* | 100% |  | 96% | 573 | 93% | 182 | 98% | 370 |
| 6 | 27634650 | 27643499 | *THUMPD1* | 80% |  | 75% | 340 | 73% | 114 | 75% | 209 |
| 6 | 27644513 | 27670891 | *Acsm4* | 94% |  | 86% | 409 | 85% | 173 | 92% | 278 |
| 6 | 27684924 | 27686135 | *nono* | 100% |  | 100% | 3034 | 100% | 2732 | 100% | 2612 |
| 6 | 27688755 | 27689305 | *LOC728643* | 100% |  | 100% | 2300 | 100% | 2764 | 100% | 2370 |
| 6 | 27893180 | 27920107 | *ACSM2A* | 77% |  | 71% | 600 | 71% | 333 | 74% | 470 |
| 6 | 27935924 | 27966023 | *ACSM5* | 66% |  | 60% | 427 | 62% | 319 | 64% | 376 |
| 6 | 27971602 | 28013520 | *PDILT* | 91% |  | 83% | 588 | 84% | 405 | 88% | 496 |
| 6 | 28019265 | 28034478 | *UMOD* | 98% |  | 93% | 768 | 95% | 645 | 98% | 708 |
| 6 | 28039975 | 28054408 | *GP2* | 98% |  | 91% | 800 | 91% | 663 | 97% | 690 |
| 6 | 28277437 | 28317406 | *GPR139* | 89% |  | 84% | 545 | 83% | 431 | 87% | 475 |
| 6 | 28440878 | 28461832 | *GPRC5B* | 97% |  | 88% | 614 | 94% | 643 | 95% | 643 |
| 6 | 28463315 | 28592455 | *IQCK* | 84% |  | 75% | 430 | 75% | 237 | 81% | 349 |
| 6 | 28595515 | 28608132 | *C16orf88* | 75% |  | 73% | 591 | 73% | 543 | 75% | 560 |
| 6 | 28622208 | 28730692 | *C16orf62* | 94% |  | 86% | 558 | 90% | 452 | 93% | 547 |
| 6 | 28740342 | 28766554 | *CP110* | 92% |  | 89% | 493 | 86% | 135 | 89% | 281 |
| 6 | 28768489 | 28786280 | *GDE1* | 95% |  | 88% | 393 | 85% | 182 | 91% | 312 |
| 6 | 28793188 | 28835777 | *TMC5* | 90% |  | 82% | 515 | 85% | 339 | 88% | 476 |
| 6 | 28899390 | 28937484 | *TMC7* | 87% |  | 77% | 385 | 80% | 306 | 83% | 344 |
| 6 | 28940812 | 28949941 | *COQ7* | 86% |  | 74% | 565 | 80% | 280 | 83% | 443 |
| 6 | 28976917 | 28983544 | *Itpripl2* | 92% |  | 88% | 501 | 92% | 366 | 90% | 491 |
| 6 | 29032381 | 29102760 | *SYT17* | 89% |  | 78% | 480 | 84% | 436 | 88% | 516 |
| 6 | 29110495 | 29126642 | *LOC728276* | 88% |  | 77% | 528 | 85% | 571 | 87% | 554 |
| 6 | 29258813 | 29336314 | *SMG1* | 94% |  | 89% | 496 | 87% | 172 | 92% | 312 |
| 6 | 29338969 | 29347302 | *ARL6IP1* | 100% |  | 99% | 495 | 99% | 213 | 100% | 276 |
| 6 | 29352515 | 29359443 | *RPS15A* | 100% |  | 100% | 959 | 99% | 553 | 100% | 695 |
